# Transient microbiota exposures activate dormant *Escherichia coli* infection in the bladder and drive severe outcomes of recurrent disease

**DOI:** 10.1371/journal.ppat.1006238

**Published:** 2017-03-30

**Authors:** Nicole M. Gilbert, Valerie P. O’Brien, Amanda L. Lewis

**Affiliations:** 1 Department of Obstetrics and Gynecology, Center for Reproductive Health Sciences, Washington University School of Medicine, St. Louis, Missouri, United States of America; 2 Department of Molecular Microbiology, Center for Women’s Infectious Disease Research, Washington University School of Medicine, St. Louis, Missouri, United States of America; University of Michigan Medical School, UNITED STATES

## Abstract

Pathogens often inhabit the body asymptomatically, emerging to cause disease in response to unknown triggers. In the bladder, latent intracellular *Escherichia coli* reservoirs are regarded as likely origins of recurrent urinary tract infection (rUTI), a problem affecting millions of women worldwide. However, clinically plausible triggers that activate these reservoirs are unknown. Clinical studies suggest that the composition of a woman’s vaginal microbiota influences her susceptibility to rUTI, but the mechanisms behind these associations are unclear. Several lines of evidence suggest that the urinary tract is routinely exposed to vaginal bacteria, including *Gardnerella vaginalis*, a dominant member of the vaginal microbiota in some women. Using a mouse model, we show that bladder exposure to *G*. *vaginalis* triggers *E*. *coli* egress from latent bladder reservoirs and enhances the potential for life-threatening outcomes of the resulting *E*. *coli* rUTI. Transient *G*. *vaginalis* exposures were sufficient to cause bladder epithelial apoptosis and exfoliation and interleukin-1-receptor-mediated kidney injury, which persisted after *G*. *vaginalis* clearance from the urinary tract. These results support a broader view of UTI pathogenesis in which disease can be driven by short-lived but powerful urinary tract exposures to vaginal bacteria that are themselves not “uropathogenic” in the classic sense. This “covert pathogenesis” paradigm may apply to other latent infections, (e.g., tuberculosis), or for diseases currently defined as noninfectious because routine culture fails to detect microbes of recognized significance.

## Author summary

Millions of women suffer from recurrent urinary tract infections (rUTI) and the only treatment option is prophylactic antibiotics, which contributes to antibiotic resistance. In experimental models, *Escherichia coli*, the dominant UTI pathogen, establishes reservoirs inside the bladder lining; it is believed that some cases of rUTI in women may be due to these reservoirs awakening in response to triggers that are still unknown. Here we present a new mouse model that demonstrates the first clinically plausible trigger of rUTI arising from these reservoirs. Specifically, we show that bladder exposure to *Gardnerella vaginalis*, a common member of the vaginal microbial community, can drive the emergence of *E*. *coli* from bladder reservoirs. Furthermore, upon its exposure to the urinary tract, this vaginal organism caused severe kidney damage and other complications, suggesting that carriage of particular vaginal bacteria could also impact a woman’s risk for kidney infection. Bladder exposure to *G*. *vaginalis* is likely to occur during sexual activity in many women. Taken together, these data provide the first explanation for why certain characteristics of the vaginal microbiota have been linked with rUTI. Finally, our findings suggest that targeting specific members of the vaginal community may be an effective strategy for treating rUTI.

## Introduction

Many disease-causing microorganisms can inhabit the human body asymptomatically, but factors that activate potentially pathogenic reservoirs to cause disease are not well understood. After an initial infection, some pathogens establish states of latency that can reactivate to cause recurrent symptomatic infections. In such cases, the problems lie not in diagnosing the recurrent infection, but rather in identifying and preventing potential triggers of reactivation and predicting which patients are at risk for developing severe manifestations of disease. An important example of bacterial latency and reactivation is bladder infection (cystitis), which has the potential to develop into life-threatening kidney and systemic disease [1–4]. The bladder is one of the most common sites of infection in humans, and urinary tract infections (UTIs) are one of the primary reasons for clinical use of antibiotics, accounting for 9% of all antibiotic use in an ambulatory care setting [5,6]. UTIs also often recur; 25% of women afflicted with acute UTI experience a recurrence within six months, and an estimated 1% of women (70 million worldwide) suffer more than six recurrent UTIs (rUTIs) each year [6]. Infection is most often caused by uropathogenic *E*. *coli*, which can establish intracellular, antibiotic-resistant bladder reservoirs in mouse models [1–3,6,7]. In humans, episodes of rUTI are often caused by the same strain of *E*. *coli* as the initial infection (~50% of cases according to whole genome sequencing and up to 82% of cases by less stringent criteria) [8]. While rUTI sometimes occurs due to reinfection of the bladder by *E*. *coli* residing in external sites (gastrointestinal tract, vagina), bladder intracellular *E*. *coli* reservoirs are regarded as another likely source of rUTI in humans [2–4,8–10]. However, clinically plausible triggers of the transition from latent bladder reservoirs to rUTI have not been identified.

Several lines of evidence implicate the vaginal microbiota in host susceptibility to bladder and kidney infections. First, women with bacterial vaginosis (BV), a dysbiosis characterized by overgrowth of fastidious anaerobes including *Gardnerella vaginalis*, are at higher risk of experiencing UTI than women with normal vaginal communities (composed mainly of *Lactobacillus* spp.) [11–14]. Second, women with rUTI experience fewer recurrences after vaginal interventions that influence the microbiota, such as administration of *Lactobacillus crispatus*, than do those receiving placebo [15,16]. Third, sexual activity is one of the strongest risk factors for both BV and UTI and is an independent risk factor for the development of ascending kidney infection and pyelonephritis, a more serious form of UTI [6,16–22]. These clinical findings support the longstanding idea that classic uropathogens gain access to the urinary tract by mechanical transfer from nearby mucosal sites, such as the vagina.

However, uropathogenic bacteria exist in mucosal sites within the context of the host microbiota, not in isolation. Thus, bacteria other than classic uropathogens like *E*. *coli* are likely to be frequently transferred from the vagina to the urinary tract. Consistent with this idea, multiple studies have isolated bacterial species from urine (*Gardnerella* and lactobacilli are among the most prominent) that are common members of the vaginal microbiota, supporting the interpretation that the bladder is likely to be regularly exposed to vaginal bacteria [23–26]. Therefore, we hypothesized that certain vaginal bacteria play an active role in the bladder, potentially triggering *E*. *coli* emergence from bladder reservoirs to cause rUTI and influencing the propensity for more serious forms of disease.

## Results

### *G*. *vaginalis* triggers *E*. *coli* rUTI

**Fig 1:**
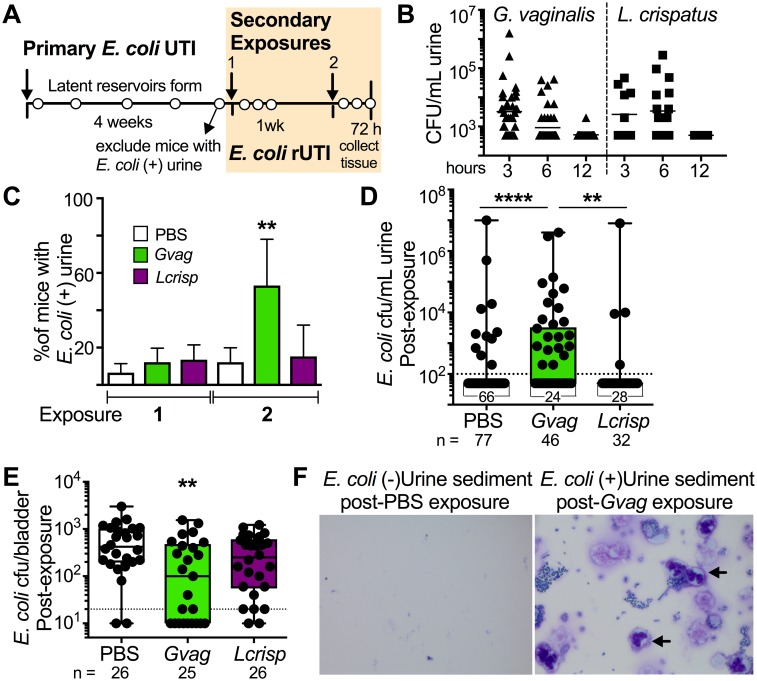
*G*. *vaginalis* bladder exposures induce *E*. *coli* emergence from latent bladder reservoirs. **(A)** Schematic of mouse model. *E*. *coli* bladder reservoirs were established during a 4 week period following a ‘Primary *E*. *coli* UTI’; those with kidney infections (bacteriuria at 4 weeks) were excluded; cream shading indicates period of exposure to *G*. *vaginalis*, *L*. *crispatus*, or PBS; open circles, urine sample collections. **(B)** *G*. *vaginalis* and *L*. *crispatus* urine titers following first exposure in mice with *E*. *coli* reservoirs. **(C)** Percentage of mice with detectable *E*. *coli* urine titers following secondary exposures. N = 6 experiments totaling 155 mice. ** *P* < 0.001 by Fisher’s exact test. **(D)** Highest *E*. *coli* urine titer from each mouse after exposure 2 (N = 6 independent experiments). Dotted line = limit of detection. A Kruskal-Wallis test was performed (*P* < 0.0001), followed by post hoc pairwise comparisons. **** *P* < 0.0001; ** *P* < 0.002 by Mann-Whitney. (**E**) *E*. *coli* reservoir titers in bladder homogenates at 72 h after exposure 2 (N = 2). ** *P* < 0.01 by Mann-Whitney. **(F)** Example of Hema-3 stained urine sediments from a PBS-exposed mouse negative for *E*. *coli* bacteriuria (left) and from a *G*. *vaginalis*-exposed mouse positive for *E*. *coli* bacteriuria (right). Arrows—neutrophils.

To test this hypothesis, we used an established model to generate mice with latent *E*. *coli* bladder reservoirs [2,27] and then investigated the effect of experimentally exposing the urinary tract to vaginal bacteria. We chose *Gardnerella vaginalis* and *Lactobacillus crispatus* to represent ‘dysbiotic’ and ‘normal’ microbiotas, respectively, as these are often dominant members of a woman’s vaginal microbiota [28,29]. Furthermore, we previously showed that *G*. *vaginalis* is sufficient to elicit clinical features of BV in a mouse vaginal infection model [30]; thus, we hypothesized that *G*. *vaginalis* may also contribute to the clinical association between BV and rUTI by stimulating the emergence of latent *E*. *coli* reservoirs. To test this idea, we transurethrally infected mice with *E*. *coli* (primary infection) and monitored their urine for *E*. *coli* bacterial titers for four weeks (Fig 1A). In C57BL/6 mice, failure to resolve urine titers after a month is nearly always indicative of kidney infection; these animals were culled from the experiment. Individuals with no detectable *E*. *coli* in urine at four weeks (~80% of those originally infected) have been previously shown to contain latent *E*. *coli* reservoirs in bladder tissue [2] and were used in subsequent experiments in which bladders were transurethrally exposed to vaginal bacteria (*G*. *vaginalis* or *L*. *crispatus*) or vehicle alone (PBS). Mice were given two such exposures one week apart, and we monitored the emergence of *E*. *coli* into urine every 24 hours for 72 hours after each exposure (Fig 1A, cream box).

Even though *G*. *vaginalis* failed to establish extended colonization of the urinary tract (Figs 1B and S1), its transient presence was sufficient to trigger *E*. *coli* emergence into urine (Fig 1C and 1D). Although *E*. *coli* was sometimes detected in urine after one exposure to *G*. *vaginalis*, the incidence was significantly higher after a second exposure (Figs 1C and S2), consistent with clinical reports linking frequency of sexual activity with increased risk of UTI [19–21]. In one experiment, two exposures with a 20-fold lower dose of *G*. *vaginalis* resulted in *E*. *coli* bacteriuria in 33% of mice, compared to 9% in a PBS control group (S3 Fig). Repeated *G*. *vaginalis* exposures leading to *E*. *coli* emergence could be spaced as far apart as one week (Fig 1) or as close as 12 hours (S3 Fig). In contrast, *E*. *coli* emergence was not induced by exposure to *L*. *crispatus*, which was cleared from the mouse urinary tract with similar kinetics to *G*. *vaginalis*, (Fig 1B–1D) or exposure to heat-killed *G*. *vaginalis* (S3 Fig). Thus, reactivation of *E*. *coli* bladder lumen infection in this model was not simply a general response to bacterial exposure. Rather, it was stimulated by a bacterium implicated in vaginal dysbiosis (*G*. *vaginalis*) [28,30] but not by a bacterium widely recognized as beneficial (*L*. *crispatus*) [15,31]. Each of these organisms commonly dominates the vaginal microbiota, supporting the physiological relevance of the monomicrobial aspect of this model.

In support of the interpretation that *E*. *coli* emerged by egress from latent bladder reservoirs, the remaining *E*. *coli* titers in the bladder tissue at sacrifice were lower in *G*. *vaginalis*-exposed mice than in those exposed to PBS or *L*. *crispatus* (Fig 1E). Finally, we found that mice in the *G*. *vaginalis*-exposed group that had high levels of *E*. *coli* bacteria in urine often also had neutrophilic infiltrates (Fig 1F), confirming that emerging *E*. *coli* constituted active rUTI. Perhaps not surprisingly, animals that went on to experience *Gardnerella*-induced rUTI had more severe *primary* infections with *E*. *coli* compared to animals exposed to *G*. *vaginalis* who did not experience rUTI (approximately 200-fold higher levels of *E*. *coli* in urine at 24 hours after the initial *E*. *coli* infection) (S4 Fig). Moreover, those that did not experience rUTI included a significantly higher proportion (17% vs 2%, *P* = 0.02 by Fishers exact) of individuals in whom *E*. *coli* bacteriuria during the initial infection was undetectable by 24 hours post infection (hpi). The lack of rUTI in these mice was not due simply to an absence of *E*. *coli* bladder reservoirs, because many mice (in both the PBS and *G*. *vaginalis* exposure groups) that cleared *E*. *coli* bacteriuria by 24 hpi still had detectable *E*. *coli* in bladder homogenates. This finding suggests that the severity and/or duration of *primary E*. *coli* UTI may be a key determinant of rUTI risk upon secondary exposure. Together, these results highlight *G*. *vaginalis* as the first clinically plausible trigger capable of reactivating latent *E*. *coli* bladder infection to cause rUTI.

### *G*. *vaginalis* causes bladder epithelial exfoliation

**Fig 2:**
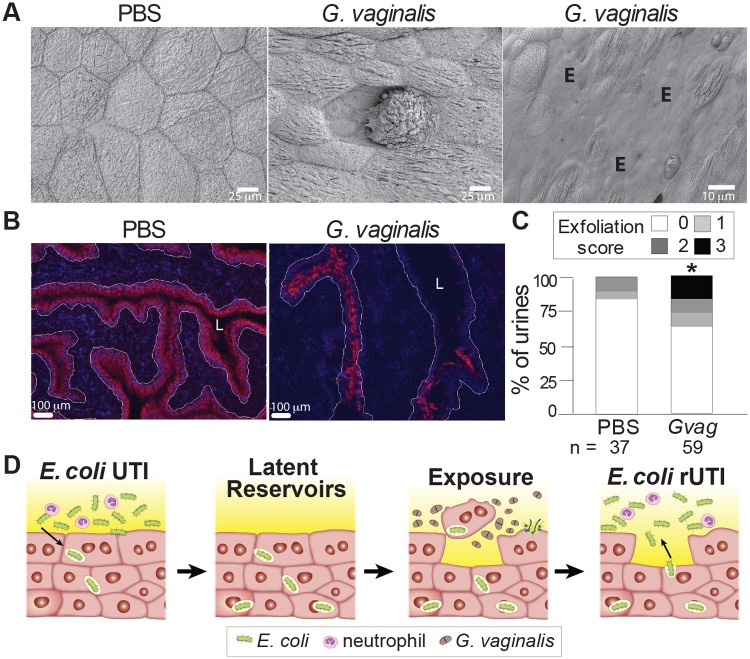
*G*. *vaginalis* causes urothelial exfoliation. (**A**) Scanning electron micrographs of splayed bladders showing the superficial hexagonal urothelial cells that line the bladder lumen. N = 2 independent experiments. PBS, n = 3; *G*. *vaginalis* n = 7. ‘E’ denotes areas of exfoliation. (**B**) Immunofluorescence microscopy of bladder sections. Red—uroplakin IIIa (superficial epithelial cells); Blue—DAPI. White lines—epithelial basement membrane; L—bladder lumen. N = 3 independent experiments; n = 2–5 mice per group. (**C**) Blinded scoring of urothelial cells in urine sediments collected between 3 and 24 h after two PBS or *G*. *vaginalis* exposures. N = 3 independent experiments; PBS, n = 37; *G*. *vaginalis*, n = 59. * *P* = 0.0374 by Fisher’s exact test. (**D**) Schematic model of *G*. *vaginalis*-induced recurrent *E*. *coli* UTI.

Because chemical exfoliation of the bladder epithelium (urothelium) in mice containing latent *E*. *coli* bladder reservoirs leads to the appearance of *E*. *coli* in urine [2,4], we wondered whether exposure to *G*. *vaginalis* also results in apoptosis and exfoliation. Scanning electron microscopy (SEM) analysis revealed that, whereas the bladder surface of PBS-exposed mice was smooth and showed contiguous hexagonal superficial urothelial cells, *G*. *vaginalis*-exposed bladders displayed patches of exfoliation and individual cells detached from their neighbors (Figs 2A and S5). This occurred in 14/14 *G*. *vaginalis* exposed mice, both with and without latent *E*. *coli* bladder reservoirs (Figs 2A and S5), but was not observed in 3 mice exposed to *L*. *crispatus* (S5 Fig). Further evidence of exfoliation comes from two additional lines of experimentation. First, at 12 hours post-exposure, animals that received *G*. *vaginalis* exhibited reduced staining for uroplakin IIIa, a marker of terminally differentiated superficial urothelial cells, compared to PBS exposed animals (Fig 2B). Second, *G*. *vaginalis*-exposed mice had higher numbers of urothelial cells in urine sediments than PBS-exposed mice (Fig 2C). Together with the established relationship between urothelial exfoliation and *E*. *coli* emergence from bladder reservoirs [2,4], these data support the conclusion that *G*. *vaginalis* triggers *E*. *coli* rUTI by inducing exfoliation (Fig 2D).

### *G*. *vaginalis* induces apoptosis in the bladder epithelium

**Fig 3:**
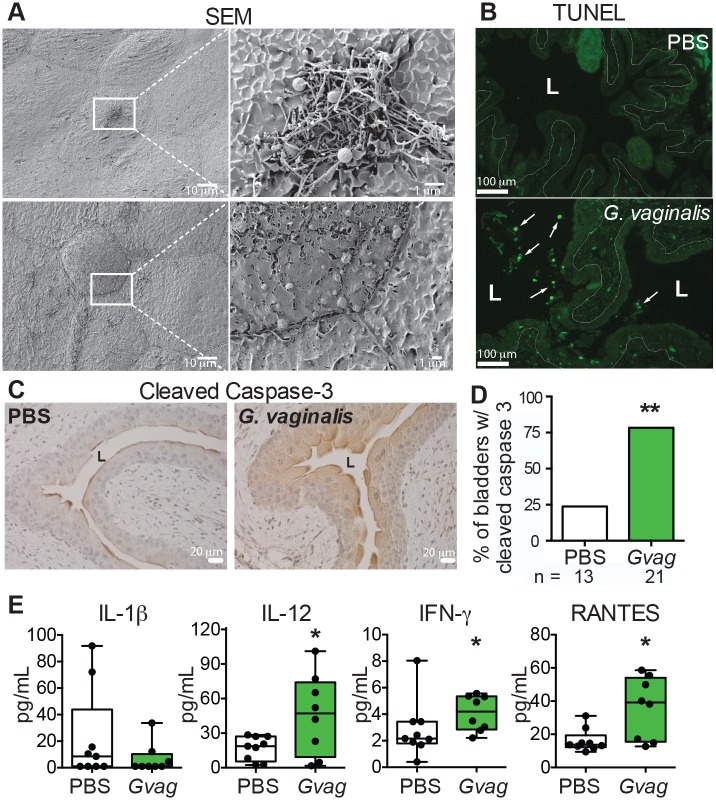
*G*. *vaginalis* induces apoptosis in the urothelium. Data are from mice containing *E*. *coli* reservoirs that were exposed twice (12 h apart) to *G*. *vaginalis* or PBS. Bladders were collected 3 h (**A**) or 12 h (**B-E**) after the second exposure. (**A**) Scanning electron microscopy (SEM) images of bladders from mice exposed to *G*. *vaginalis*. Top: *G*. *vaginalis* association with membrane protrusions. Bottom: membrane blebbing consistent with apoptotic body formation. (**B**) TUNEL staining of bladder sections revealed TUNEL-positive cells (white arrows) within the lumen (L) and superficial urothelium of *G*. *vaginalis*-exposed bladders. White dotted line denotes the epithelial basement membrane. (**C**) Immunohistochemistry of bladder sections stained for cleaved caspase-3 (brown). (**D**) Percentage of bladders in each exposure group that stained positively for cleaved caspase-3. N = 2 independent experiments, 2–5 mice per group. (**E**) Level of pro-inflammatory cytokines in bladder homogenates. A D’Agostino & Pearson omnibus normality test was performed followed by appropriate pairwise analysis (either unpaired t-test or Mann-Whitney). * *P* < 0.05.

Previous studies have implicated programmed cell death in urothelial exfoliation [32] [9]. Consistent with apoptosis, we observed membrane protrusions [33] and blebbing in scanning electron microscopy (SEM) images of all bladders exposed to *G*. *vaginalis* but in none of the SEM images from mice exposed to PBS (Figs 3A and S6). *G*. *vaginalis* also caused membrane protrusions and blebbing in mice that were not given a primary *E*. *coli* infection and thus had no *E*. *coli* bladder reservoir (S6 Fig). In further support of the idea that *G*. *vaginalis* exposure caused apoptosis, bladder epithelial cells from mice that were exposed to *G*. *vaginalis* were TUNEL positive (Fig 3B) and displayed pronounced staining for cleaved caspase-3 (Figs 3C and 3D and S7), both markers of apoptosis.

Consistent with apoptosis as an immunologically silent form of cell death, bladders of *G*. *vaginalis*-exposed mice showed no increase in the levels of the pro-inflammatory cytokine IL-1β (Figs 3E and S8), a marker of bladder epithelial exfoliation via pyroptosis [32]. In contrast, levels of IL-12, IFN-γ, and RANTES were higher in the bladder upon exposure to *G*. *vaginalis* than upon exposure to PBS (Figs 3E and S8). This cytokine signature is consistent with bladder exfoliation, as these cytokines are also induced as a response to stripping of the bladder epithelium by the immunotherapeutic agent *Mycobacterium bovis* bacillus Calmette-Guerin (BCG) [34]. Together, these results demonstrate that bladder exposure to *G*. *vaginalis* results in apoptosis of the urothelium. Evidence that this mechanism might also occur in humans comes from clinical findings that bladder biopsies from women prone to rUTI exhibit more urothelial apoptosis than do those from controls [35].

### *G*. *vaginalis* causes kidney inflammation and injury and predisposes to severe *E*. *coli* infection

**Fig 4:**
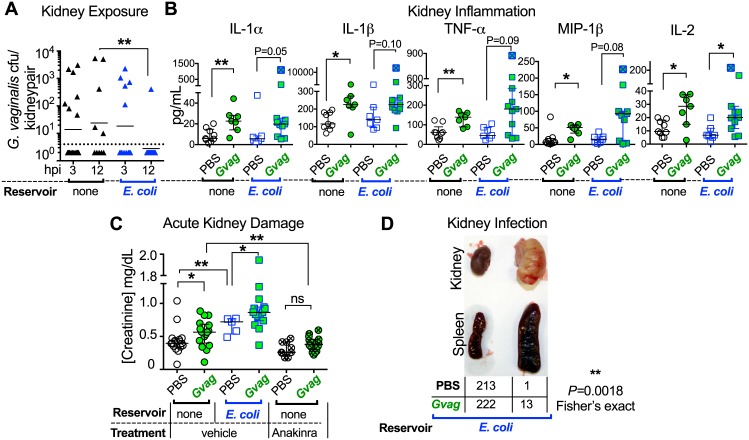
*G*. *vaginalis* causes IL-1 receptor-mediated kidney injury and increases the incidence of severe *E*. *coli* kidney and systemic infection. (**A-C**) Data are from mice that were exposed twice (12 h apart) to PBS or *G*. *vaginalis* as described in S3 Fig. (**A**) *G*. *vaginalis* titers in kidney tissue. (**B**) Cytokine/chemokine levels in kidney homogenates at 12 h after the second exposure. Kruskal-Wallis tests were performed followed by post hoc pairwise comparison. For comparison, data from an abscessed kidney collected at 72 h after exposure (not included in the statistical analysis) are denoted by symbols with a blue ‘X’. ** *P* < 0.005, * *P* < 0.05, Mann-Whitney (uncorrected *P* values). (**C**) Serum creatinine levels, a marker of acute kidney injury, at 12 h after the second bladder exposure. Mice received 2 intraperitoneal injections of PBS (vehicle) or anakinra at ~16h prior to and at the time of transurethral inoculation. N = 6 independent experiments. ** *P* < 0.005, * *P* < 0.05. (**D**) Incidence of *E*. *coli* kidney infection with abscessed kidney and splenomegaly at 72 h post exposure. Data are compiled from 12 experiments with two exposures (12 h and 1 wk apart, including mice injected with PBS as in C) and 1 experiment with 3 exposures, each 24 h apart. ** *P* = 0.0018, Fisher’s exact.

Reactivation of latent *E*. *coli* bladder infection has the potential to lead to a more severe manifestation of UTI: ascending infection of the kidneys (pyelonephritis), which can lead to urosepsis and death. Pyelonephritis often occurs in patients with associated risk factors such as catheterization, urinary tract obstruction, or other anatomical abnormalities. However, in otherwise healthy women, many of the identified risk factors of pyelonephritis overlap with BV risk factors (e.g. sexual activity, new sex partner[s], lifetime number of sexual partners, oral sex, spermicide use, smoking, and vaginal douching) [22,36]. Therefore, we used our model to test the hypothesis that urinary tract exposures to *G*. *vaginalis* could influence the development of kidney infection by *E*. *coli*. First, we detected *G*. *vaginalis* in 30–40% of kidneys at levels of 50–3000 cfu per kidney pair at three hours post-exposure both in mice with and without *E*. *coli* reservoirs (Figs 4A and S1). *G*. *vaginalis*-exposed mice displayed higher levels of IL-1α, IL-1β, TNF-α, MIP-1β, and IL-2 in the kidney (Fig 4B) and higher levels of serum creatinine, a biomarker of acute kidney injury [37], than PBS controls (Fig 4C). Finally, we examined the relationship between kidney inflammation and injury, demonstrating that treatment of naïve mice (no *E*. *coli* reservoirs) with an interleukin-1-receptor antagonist (anakinra) prior to and during *G*. *vaginalis* exposure blocked kidney injury (Fig 4C). In summary, *G*. *vaginalis* was sufficient to cause kidney damage independent of the presence of *E*. *coli* and this phenotype could be blocked by an IL-1-receptor antagonist.

In addition to the inflammation and injury caused by *G*. *vaginalis* in the kidney (Fig 4B and 4C), exposure to the bacterium also rendered the kidney more susceptible to severe *E*. *coli* infections. Approximately 6% (13/235) of *G*. *vaginalis*-exposed animals containing *E*. *coli* reservoirs developed high titer *E*. *coli* kidney infection with severe abscesses and splenomegaly (Fig 4D). This phenotype was extremely rare among reservoir-containing animals exposed to PBS across all experiments we performed (1/214, ~0.5%), and was never seen in naïve mice exposed only to *G*. *vaginalis*. The low incidence of abscesses in our model echoes the rarity of pyelonephritis in women. Although only 1% of healthy reproductive-age women with cystitis go on to experience pyelonephritis [6], one study showed that >9% of patients with *G*. *vaginalis* in urine had pyelonephritis [38]. As would be expected, pro-inflammatory cytokines/chemokines were present at even higher levels in an abscessed kidney collected at 72 h after exposure (Fig 4B ‘X’ symbols). Analysis of spleens collected from a subset of mice with abscessed kidneys revealed that both *G*. *vaginalis* and *E*. *coli* were capable of causing systemic infection. In contrast, neither organism was detected in 20/20 spleens from mice lacking abscesses and splenomegaly (*P* = 0.0119, Fisher’s exact). Notably, *G*. *vaginalis* has been found in systemic infections in humans [39–42]. Our findings are also consistent with a clinical study in which inpatients with *G*. *vaginalis* in urine were more likely to have a history of rUTI and current kidney infection than patients without *G*. *vaginalis* in urine [38]. Taken together, our results demonstrate that *G*. *vaginalis* exposure enhances *E*. *coli* urinary tract pathogenesis in the mouse model; whether this also occurs in women warrants further investigation.

## Discussion

**Fig 5:**
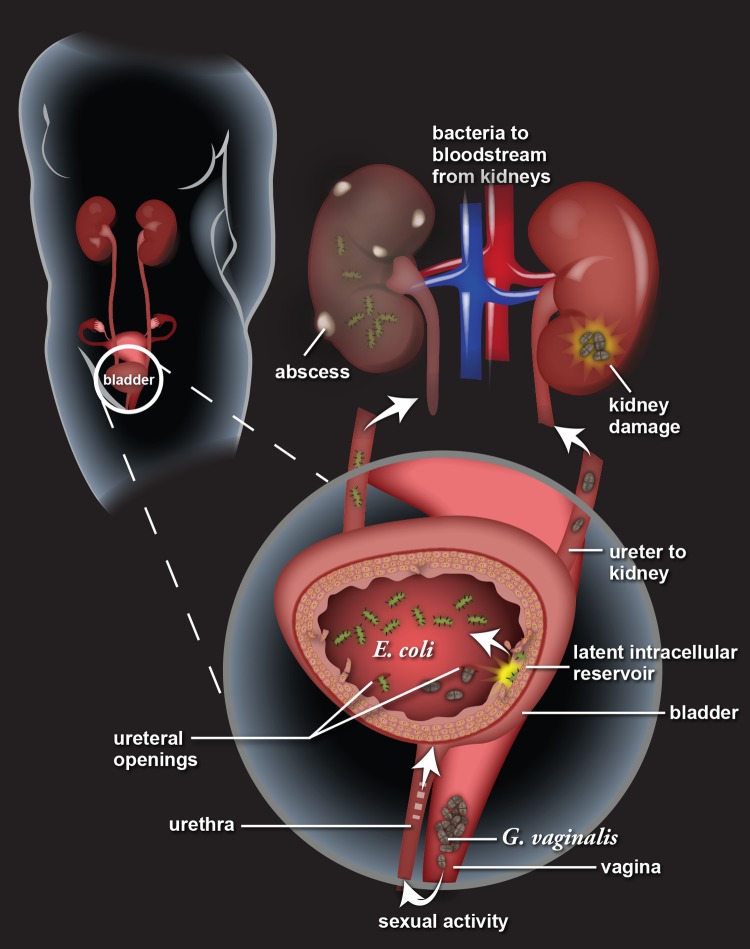
Model of *Gardnerella vaginalis* “covert pathogenesis.” In women, approximately half of recurrent urinary tract infection (rUTI) episodes are caused by an *E*. *coli* strain identical to the strain that caused the initial infection. Here we present a model of one mechanism of rUTI: activation of latent intracellular *E*. *coli* reservoirs in the bladder. *G*. *vaginalis* urinary tract exposure, likely a consequence of sexual activity, results in exfoliation of the bladder epithelium and damage to the kidney. Subsequent to exfoliation, *E*. *coli* emerges from intracellular reservoirs into the bladder lumen, where it can ascend into the kidney, sometimes causing severe inflammation and systemic infection. Often *E*. *coli* rUTI occurs after *G*. *vaginalis* clearance from the urinary tract. These findings have important implications for our understanding of rUTI etiology and point to *G*. *vaginalis* colonization as a potentially important marker of rUTI risk.

Latent *E*. *coli* reservoirs are regarded as a source of rUTI, but until now, clinically relevant triggers of reactivation have been unknown. Here, we used a new mouse model to show that transient exposures to *G*. *vaginalis*, a prevalent member of the vaginal microbiota and a dominant species in women with BV, is sufficient to drive *E*. *coli* emergence from reservoirs and the development of more serious manifestations of UTI, such as severe kidney infection (see model in Fig 5). Our study both shows a direct role for the vaginal microbiota in UTI pathogenesis and provides a mechanistic understanding of the cellular events leading to rUTI. We identified a single bacterial species that may explain the long-standing but poorly understood clinical association between BV and UTI. We note two possible translational outcomes of this finding. First, our data suggest that therapies aimed at reducing *G*. *vaginalis* colonization of the vagina could protect against *E*. *coli* rUTI. Second, given our finding that *G*. *vaginalis* exposures cause kidney damage and increased the likelihood of kidney and systemic infections by *E*. *coli* in mice, *G*. *vaginalis* colonization could be a risk factor for pyelonephritis in women. The possibility of preventing rUTI and subsequent pyelonephritis and systemic infection by targeting *G*. *vaginalis* is an exciting concept given the alarming global rise in multi-drug resistant *E*. *coli* [43].

Our model provides a new lens through which to view clinical findings relevant to the urinary tract. For instance, it is widely thought that the strong relationship between sexual activity and the risk of UTI [19–21] exists because *E*. *coli* or other classical uropathogens are transferred to the bladder from surrounding niches, such as the vagina. Our data suggest an alternative, albeit not mutually exclusive, model in which urinary tract exposure to certain members of the vaginal microbiota, such as *G*. *vaginalis*, can elicit *E*. *coli* emergence from reservoirs *within* the bladder. On this note, we emphasize that vaginal colonization with *G*. *vaginalis* can occur in women who do not meet the diagnostic criteria for BV (for example, see the table of strains and BV status in [44]). However, the finding that women with BV generally have high vaginal burdens of *G*. *vaginalis* may make urinary tract exposure upon sexual activity (and the potential consequences thereof) all the more likely. Previous reports that vaginal administration of *L*. *crispatus* reduces the incidence of subsequent rUTI have suggested that *L*. *crispatus* may act by reducing the vaginal reservoir of *E*. *coli* [15]. In light of our data, we propose an additional potential explanation for the protective effect of probiotic *L*. *crispatus*: the displacement of *G*. *vaginalis* from the vagina, thereby reducing the likelihood of *G*. *vaginalis*-induced recurrent *E*. *coli* UTI. Taken together, these observations suggest that repeated transient exposures to *G*. *vaginalis* may be broadly important in determining susceptibility to *E*. *coli* rUTI and may act through previously unrecognized routes.

Our results shed light on established, albeit largely ignored, clinical findings of *G*. *vaginalis* in both the bladder and the kidney [45–51]. With respect to the bladder, our discovery that *G*. *vaginalis* causes damage to the urothelium are relevant to a study in which 25% of women with urinary frequency or dysuria had urine samples containing *G*. *vaginalis* in counts greater than 10^3^ cfu/mL. Furthermore, *G*. *vaginalis* was isolated more frequently as a member of the “urinary microbiome” of women suffering from urgency urinary incontinence (a.k.a. overactive bladder syndrome) than healthy controls [23]. Although there is some debate regarding the impact of “urinary microbiome” bacteria and whether or not they represent an established community, our findings emphasize that long-standing colonization of the bladder may not be required for bacteria such as *G*. *vaginalis* to affect disease outcomes. In regards to the kidney, our finding that *G*. *vaginalis* was associated with markers of kidney injury parallel a study in which *G*. *vaginalis* was detected in urine collected directly from the bladder in 33% of patients with “sterile pyelonephritis” (i.e., patients with reflux scarring in the apparent absence of bacterial infection) [45]. Strikingly, *G*. *vaginalis* was localized to the upper urinary tract in 75% of these patients [45]. Despite these reports of *G*. *vaginalis* in the urinary tract, the classification of *G*. *vaginalis* as a “uropathogen” is currently controversial. We note that fastidious growth requirements make *G*. *vaginalis* unrecoverable, or at least unidentifiable, under conditions most often used by clinical microbiology labs for the culture and identification of potential uropathogens. Indeed, a recent investigation of 120 patient urines identified *G*. *vaginalis* by proteomic analysis in nearly 20% of samples, none of which were classified as containing *G*. *vaginalis* based on standard aerobic culture [52]. Thus, it is likely that the incidence of *G*. *vaginalis* in the urinary tract is underestimated. Taken together with this body of clinical findings, our data implicate *G*. *vaginalis* as a cause of urologic pathology, both together with and independent of *E*. *coli*, and strongly support further investigations into the possible role of this organism in urologic conditions of unknown or disputed etiology. Also of note, it is becoming increasingly evident that there is a broad range of genetic and phenotypic diversity among individual strains of *G*. *vaginalis* [53–55]. Future studies will reveal whether certain lineages of *G*. *vaginalis* are more frequently associated with negative effects in the urinary tract.

Finally and more broadly, our work suggests a new paradigm for understanding and diagnosing recurrent infections. With few exceptions, the diagnosis and treatment of bacterial infections is founded on the assumption that the pathogen present at the time of clinical presentation is the main driver of disease. However, this classical view of pathogenesis may be overly simplistic. Our work provides a new paradigm we term “covert pathogenesis”, whereby transient exposure to organism A triggers disease caused by organism B, despite organism A being absent at the time of disease emergence. These short-lived bacterial exposures are likely to be missed in the clinical setting because they occur before disease symptom onset but nevertheless could drive both the recurrence and severity of disease caused by a recognized pathogen. “Covert pathogenesis” may be relevant in other contexts in which the natural triggers of disease have remained obscure. For instance, we see several striking parallels between our model and another extremely common latent bacterial infection: tuberculosis (TB). Approximately one-third of the world’s population harbors latent TB in the lungs, with 5–15% developing symptomatic disease [56]. The recent reports of the “lung microbiome” [57,58] echo the concept that the lung may be routinely transiently exposed to bacteria, and thus “covert pathogenesis” should be explored as a potential mechanism of reactivation of latent TB. Ultimately, our finding that short-lived microbial exposures influence disease pathogenesis may have far-reaching implications beyond the urinary tract.

## Materials and methods

### Bacterial strains and growth conditions

Uropathogenic *Escherichia coli* clinical cystitis isolate UTI89 containing a kanamycin resistance cassette (UTI89 kan^R^) [59] was grown static at 37°C under aerobic conditions in Lysogeny broth (LB) medium or on LB agar plates supplemented with 25 μg/mL kanamycin. A spontaneous streptomycin-resistant (Sm^R^) strain of *Gardnerella vaginalis* derived from clinical isolate JCP8151B (from a BV-positive woman) was cultured in NYCIII media or on NYCIII agar plates supplemented with 1 mg/mL streptomycin at 37°C in an anaerobic chamber. *Lactobacillus crispatus* GED7756A was obtained from a vaginal swab of a BV-negative woman (Nugent score = 1). The vaginal swab was transported from the clinic to the lab using Port-A-Cu pre-reduced anaerobic transport media tubes (BD). Tubes were brought into a vinyl anaerobic airlock chamber (Coy Products) under an atmosphere maintained at approximately 1% hydrogen and 0 ppm oxygen. Within 24 hours the swab was streaked to isolation using de Man Rogosa and Sharpe (MRS) medium. All growth incubations were static at 37°C, under aerobic (*E*. *coli*) or anaerobic (*G*. *vaginalis* and *L*. *crispatus*) conditions, as we have previously described [30,44,60,61]. For infection experiments, *E*. *coli* was inoculated from an LB agar plate into 10 mL LB medium, grown ~24 h, then subcultured 1:10 in fresh LB for 18 h. *E*. *coli* inoculum was prepared in phosphate buffered saline (PBS) at OD 0.35 (~1–2 x 10^7^ colony forming units (cfu) in 50 μL). *G*. *vaginalis* was inoculated from an NYCIII agar plate into 3 mL NYCIII media and grown overnight, ~18 h. *G*. *vaginalis* inoculum was prepared in PBS at OD 5 (~5 x 10^7^–1 x 10^8^ cfu in 50 μL). *L*. *crispatus* was inoculated from an MRS agar plate into 3 mL MRS media and grown overnight ~18 h. *L*. *crispatus* inoculum was prepared in PBS at OD 10 (~1 x 10^8^ cfu in 50 μL). For experiments using heat-killed *G*. *vaginalis*, the inoculum was divided into two aliquots and one was incubated at 80°C for 10 min. Complete killing was confirmed by incubation on NYCII medium for 48 h anaerobically at 37°C.

### Ethics statement

Mouse experiments were carried out in strict accordance with the recommendations in the Guide for the Care and Use of Laboratory Animals. The protocol was approved by the Animal Studies Committee of Washington University School of Medicine (Protocol Number: 20140114).

### Mice

Five- to six-week old female C57BL/6 mice were obtained from Charles River Labs (NCI grantee order from Fredericks facility). Mice were given a regular chow diet in a specific pathogen-free facility at Washington University in St. Louis School of Medicine.

### Mouse infection/exposure model

Mice (6–8 weeks of age) were anaesthetized with isofluorane and then inoculated transurethrally with 10^7^ cfu *E*. *coli* (UTI89 kan^R^) in 50 μL sterile PBS as previously described [60,61]. Urine was collected and monitored for *E*. *coli* titers at 24 hours post infection (hpi) and weekly for 4 weeks. Mice with *E*. *coli* bacteriuria (any level of bacteria in urine) at 4 wpi, thus presumed to have *E*. *coli* kidney infection based on previous reports [62] and our own observations, were culled from the experiments. Mice that were negative for bacteriuria, thus presumed to contain intracellular *E*. *coli* reservoirs, were divided into groups for transurethral exposure to either PBS, *G*. *vaginalis* (7x10^7^- 1x10^8^ JCP-8151B Sm^R^) or *L*. *crispatus* (1x10^8^ GED7756A). For individual experiments, 20 mice per group were required, to be powered (0.8) to detect a statistically significant difference (p<0.05) between a 10% rate of spontaneous rUTI in the control group and 50% rate of rUTI in the *G*. *vaginalis* group. The groups were frequency matched based upon the time course of clearance of *E*. *coli* urine titers during the initial infection (also, *E*. *coli* titers at 24 hours post initial infection were not statistically different between groups). Mice were inoculated transurethrally with PBS, *G*. *vaginalis* or *L*. *crispatus* twice (12 h or 1 w apart). Urine was collected at 3, 6, 12, 24, 48 and 72 hpi (as indicated in text and figure legends) and bacterial titers enumerated by serial dilution and plating on selective media for either *E*. *coli* (LB+kanamycin) or *G*. *vaginalis* (NYCIII+streptomycin). Mice were humanely sacrificed (cervical dislocation under isofluorane anaesthesia) to aseptically harvest bladders, kidneys and spleens. Homogenates were prepared in 1 mL sterile PBS and plated on appropriate selective media. Bacterial burden in each sample was calculated as cfu/bladder or cfu/kidney pair. Samples with no colonies were plotted at one-half of the limit of detection. A 500 μL aliquot of each bladder and kidney homogenate was centrifuged at 13,000 rpm in a benchtop microcentrifuge for 5 minutes at 4°C and supernatants were removed and stored at -20°C for cytokine analysis, as described below.

### Urine sediment microscopy

Eighty microliters of a 10-fold dilution of urine was cytospun onto coated Cytopro Dual microscope slides and stained with a Hema 3 staining kit (Fisher) to visualize epithelial cells and neutrophils (PMNs). Slides were observed on a Olympus Vanox AHBT3 microscope. Urine samples were scored qualitatively for the degree of epithelial exfoliation (ranging from 0 = none to 3 = robust) in a blinded fashion.

### Scanning electron microscopy

Bladders were aseptically harvested, splayed, and fixed in EM fixative (2% paraformaldehyde, 2% glutaraldehyde in 0.1M sodium phosphate buffer, pH 7.4). Samples were prepared by critical point drying. Briefly, samples were post-fixed in 1.0% osmium tetroxide, dehydrated in increasing concentrations of ethanol, then dehydrated at 31.1°C and 1072 PSI for 16 minutes in a critical point dryer. Samples were mounted on carbon tape-coated stubs and sputter-coated with gold/palladium under argon. Bladders shown in Figs 2, 3 S5 and S6 were imaged on a Zeiss Crossbeam 540 FIB-SEM. For preliminary analysis samples were imaged on a Hitachi S-2600H SEM. ImageJ 1.49v (National Institutes of Health, USA) was used to add scale bars.

### Immunofluorescence and immunohistochemistry

Bladders were fixed overnight in methacarn (60% methanol, 30% chloroform, 10% glacial acetic acid) at room temperature followed by paraffin embedding and histological slide preparation performed by the Washington University School of Medicine Histology Core. For immunofluorescence, slides were deparaffinized, hydrated, blocked with 1% BSA and 0.3% triton X-100 in PBS, incubated with primary antibody to uroplakin III (M-17 goat polyclonal IgG, Santa Cruz Biotechnology) in blocking buffer overnight at 4°C and secondary antibody in PBS for 30–60 minutes at room temperature. Antibodies are verified at 1DegreeBio (http://1degreebio.org/). Samples were stained with Hoechst for 5 min, mounted with ProLong Gold anti-fade mounting medium (ThermoFisher / Life Technologies) and then visualized on an Olympus BX61 fluorescent microscope using SlideBook 5.0 software. Terminal deoxynucleotidyl transferase dUTP nick end labeling (TUNEL) staining was performed using the *In situ* Cell Death Detection Kit (Roche) following manufacturer’s protocol for paraffin-embedded tissue. Immunohistochemistry analysis of cleaved caspase-3 was performed using SignalStain Apoptosis (Cleaved Caspase-3) IHC kit (Cell Signaling Technology) according to the manufacturer's protocol.

### Cytokine/chemokine analysis

Tissue homogenate supernatants collected as described above were thawed on ice, centrifuged again at 4°C to remove any remaining particulates and then cytokine expression was measured using the Bio-Plex-Pro Mouse Cytokine 23-Plex Panel multiplex cytokine bead kit (Bio-Rad), which quantifies 23 different cytokines/chemokines. Assay was performed according to manufacturer instructions, except using 10-fold less standard and half the amount of coupled beads and detection antibodies indicated in the protocol.

### Serum analysis of kidney damage

Blood was collected from mice at sacrifice by cardiac puncture and put into 400-μl Microtainer serum separation tubes (BD). After coagulation, Microtainer tubes were subjected to centrifugation at 15,000 × g for 5 min at room temperature and stored at −20°C. Serum was thawed on ice and creatinine levels were measured using the QuantiChrom Creatinine Assay Kit (BioAssay Systems) according to manufacturers’ instructions.

### Statistics

The figures show individual data with each data point from a different animal with a line at the geometric mean or with box and whiskers (Min. to Max.) plots. The statistical tests used to analyze each set of data are indicated in the figure legends. For non-parametric analyses, differences between the experimental groups were analyzed with a two-tailed unpaired Mann-Whitney U test or Kruskal-Wallis test for comparisons of more than two groups using Prism GraphPad software. For analysis of cytokines, raw uncorrected *P* values are provided.

## Supporting information

S1 Fig *G*. *vaginalis* is rapidly cleared from the mouse urinary tract. Graphs show titers of *G*. *vaginalis* recovered from naïve mice not previously infected with *E*. *coli* (open symbols) or *E*. *coli* reservoir-containing mice (closed symbols) in (**A**) urine (**B**) bladder homogenates and (**C**) kidney homogenates at the indicated time points following a first or second bacterial exposure. While it is unclear how *G*. *vaginalis*, which is non-motile, accesses the kidney, interestingly there is precedent for non-motile bacteria accessing the kidney in C57BL/6 mice: *Staphylococcus saprophyticus* [63], *Enterococcus faecalis* [64] and Group B *Streptococcus* (GBS) [65]. Thus, this phenomenon is not limited to *G*. *vaginalis* and could be a shared feature of Gram positive bacterial infection of the urinary tract. Data from 3 and 12 h after exposure 2 are also shown in Fig 4A. * *P* < 0.05; ** *P* < 0.01, Mann-Whitney. Dotted line = limit of detection.
(EPS)

S2 Fig *E*. *coli* emergence into urine following exposure to *G*. *vaginalis*. Exposures 1 and 2 were 1 week apart, as indicated in Fig 1A. Dotted line = limit of detection. Samples with no detectable bacteria are staggered below the limit of detection. N = 6 independent experiments.
(TIFF)

S3 Fig Exposure to *G*. *vaginalis* triggers *E*. *coli* emergence from bladder reservoirs. **(A)** *E*. *coli* emergence following PBS (n = 11) or *G*. *vaginalis* (n = 12) exposure. Experiment was performed as outlined in Fig 1A, using 5 x 10 ^6^ cfu *G*. *vaginalis*, a 20-fold lower dose than that used in Fig 1. **(B)** Schematic of experiments shown in C-D, which were performed as outlined in Fig 1A, except that the two *G*. *vaginalis* (or PBS control) exposures were given 12 h apart (instead of 1 week). Additionally, two experiments included a group of mice exposed to heat-killed *G*. *vaginalis*. **(C)** Percentage of mice with detectable *E*. *coli* urine titers post exposure (N = 5 experiments totaling 184 mice). ** P < 0.001 by Fisher’s exact test. **(D)** Highest *E*. *coli* urine titers 24–72 h after 2 exposures. A Kruskal-Wallis test was performed (P = 0.002), followed by post hoc pairwise comparisons. ** P < 0.003, * P < 0.05 by Mann-Whitney. Dotted line denotes the limit of detection.
(EPS)

S4 Fig *E*. *coli* emergence from bladder reservoirs is more common in mice that experienced high titer *E*. *coli* primary infection. *E*. *coli* titers at 24 hpi during the primary infection phase (represented by the first open circle in Fig 1A). Data are from the same 6 independent experiments depicted in Fig 1D and 1E. (**A**) Comparison of the *E*. *coli* infection level in mice that were later exposed to either PBS or *G*. *vaginalis* (Fig 1A, green box). The distribution of initial (24 h after inoculation) *E*. *coli* titers was not significantly different between the two groups prior to *G*. *vaginalis* exposure. (**B**) The same data as shown in (**A**), except stratified based upon whether the mouse experienced *E*. *coli* emergence into urine following *G*. *vaginalis* exposure. *E*. *coli* emergence from reservoirs was contingent upon initial 24 hpi *E*. *coli* urine titers >10^4^ cfu/mL, Fisher’s exact *P* = 0.0053. A Kruskal-Wallis test was performed, followed by post-hoc pairwise comparisons. ** *P* < 0.01, Mann-Whitney U test. In both panels the limit of detection was 100 cfu.
(EPS)

S5 Fig *G*. *vaginalis* induces urothelial exfoliation independent of latent *E*. *coli* infection. Scanning electron microscopy of splayed bladders from naïve (no *E*. *coli* reservoirs) (**A**) and *E*. *coli* reservoir-containing (**B**) mice exposed twice (12 h apart) to PBS, *G*. *vaginalis* or *L*. *crispatus*, as indicated, and harvested 3 hours after the second exposure. This phenotype of epithelial exfoliation was observed in two independent experiments with mice containing *E*. *coli* reservoirs (7/7 *G*. *vaginalis*-exposed mice, 0/3 PBS-exposed mice, 0/2 *L*. *crispatus*-exposed mice) and in three independent experiments with naive mice (7/7 *G*. *vaginalis*-exposed mice, 0/3 PBS-exposed mice, 0/1 *L*. *crispatus*-exposed mice). ‘**E**’ denotes areas of exfoliation.
(TIF)

S6 Fig *G*. *vaginalis* exposure induces urothelial membrane protrusions and blebbing independent of latent *E*. *coli* reservoirs. Scanning electron microscopy (SEM) of splayed bladders from control mice exposed to PBS (**A-C**), or from naïve (no *E*. *coli* reservoirs) (**D-F**) and *E*.*coli* reservoir-containing (**G-O**) mice exposed twice to *G*. *vaginalis* (12 h apart) and harvested 3 hours after the second exposure. Black arrows denote areas of membrane protrusions (~100 nm diameter) and blebbing. Panel **M** shows a red blood cell, seen in one bladder exposed to *G*. *vaginalis*. Images are representative of two independent experiments with mice containing *E*. *coli* reservoirs (phenotype was observed in 7/7 *G*. *vaginalis*-exposed mice and 0/3 PBS-exposed mice) and three independent experiments with naive mice (phenotype was observed in 7/7 *G*. *vaginalis*-exposed mice and 0/3 PBS-exposed mice).
(TIF)

S7 Fig Immunohistochemistry for cleaved caspase-3 in bladder tissue sections. Additional images from experiments described in Fig 2C. Top box: bladders from naïve mice (no *E*. *coli* reservoirs). Bottom box: bladders from mice containing *E*. *coli* reservoirs. Images within each dotted line box are from bladder sections that were on the same slide. Scale bars are 20 μm, with the exception of the top row of images from *E*. coli reservoir-containing bladder are low magnification (scale bar 100 μm), with white dotted boxes denoting the area at higher magnification in the image directly below. Box in the bottom right corner shows the number of mice in each group that were either (-) or (+) for cleaved caspase-3 staining based on blinded scoring, as described in Materials and Methods. These values were used to generate the graph shown in Fig 2D.
(TIF)

S8 Fig Cytokine/Chemokine analysis of bladder tissue. Naive mice (open symbols) or *E*. *coli* reservoir-containing mice (closed symbols) exposed to either PBS (circles) or *G*. *vaginalis* (squares). A Kruskal-Wallis test detected significant difference between the groups for RANTES, IL-12 and IFN-γ. A D’Agostino-Pearson omnibus normality test was performed, followed by appropriate post-hoc pairwise analysis (either unpaired t-test or Mann-Whitney U test). ** *P* = 0.01; * *P* < 0.05. Unless otherwise depicted with a line, *P* values represent statistically significant differences from naive mice exposed to PBS (open circles).
(TIFF)
