# Supplementary figures and images for: Transient microbiota exposures activate dormant Escherichia coli infection in the bladder and drive severe outcomes of recurrent disease
Source: PLoS Pathog. 2017 Mar 30;13(3):e1006238. doi: 10.1371/journal.ppat.1006238 (PMC5373645; doi:10.1371/journal.ppat.1006238)

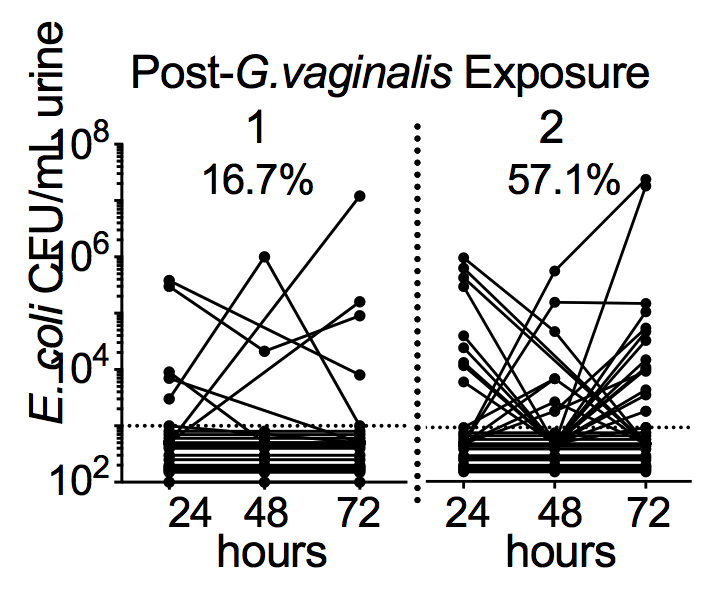

Supplement: S2 Fig — Exposures 1 and 2 were 1 week apart, as indicated in Fig 1A. Dotted line = limit of detection. Samples with no detectable bacteria are staggered below the limit of detection. N = 6 independent experiments. (TIFF) [file ppat.1006238.s002.tiff]

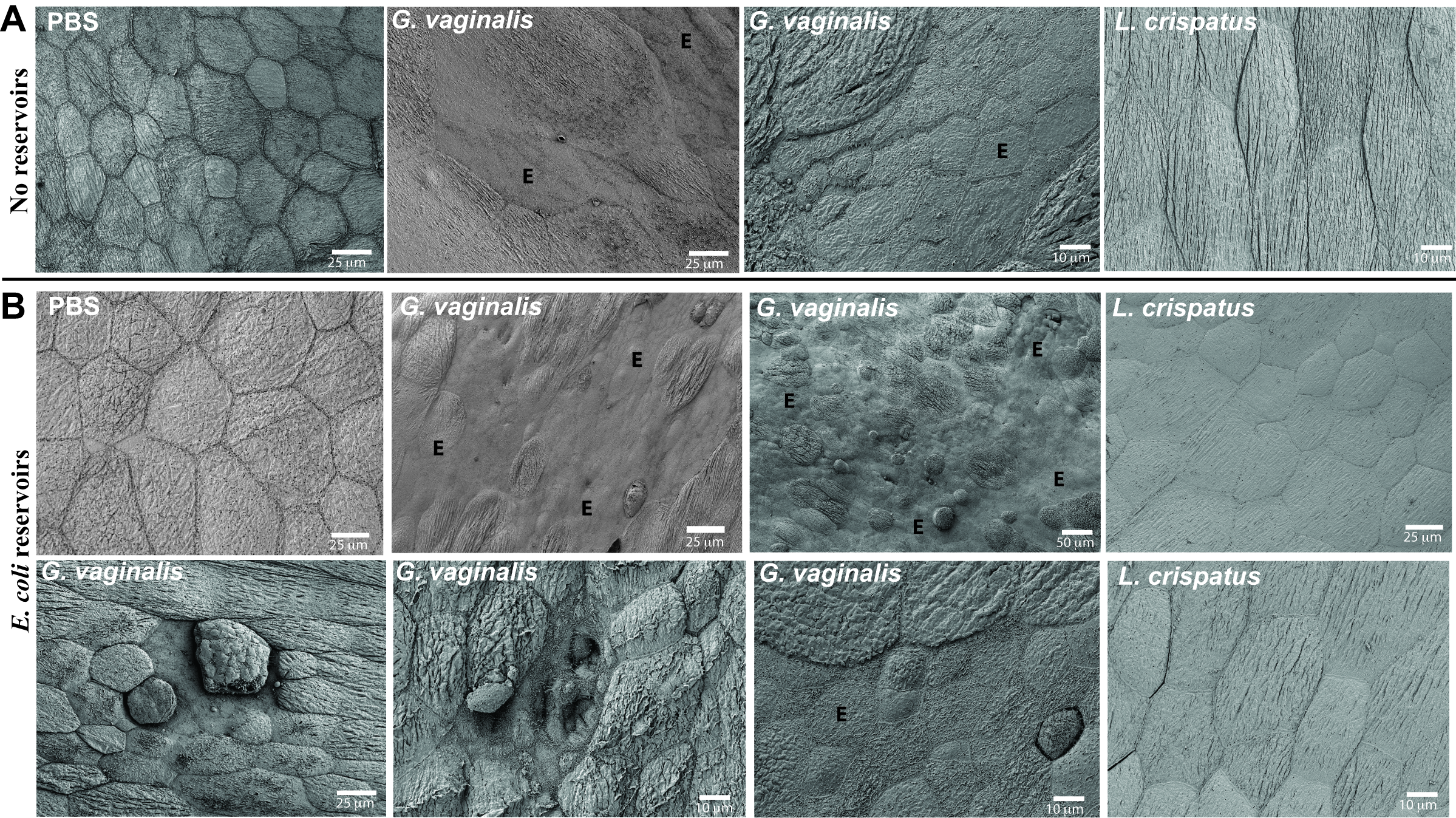

Supplement: S5 Fig — Scanning electron microscopy of splayed bladders from naïve (no E. coli reservoirs) (A) and E. coli reservoir-containing (B) mice exposed twice (12 h apart) to PBS, G. vaginalis or L. crispatus, as indicated, and harvested 3 hours after the second exposure. This phenotype of epithelial exfoliation was observed in two independent experiments with mice containing E. coli reservoirs (7/7 G. vaginalis-exposed mice, 0/3 PBS-exposed mice, 0/2 L. crispatus-exposed mice) and in three independent experiments with naive mice (7/7 G. vaginalis-exposed mice, 0/3 PBS-exposed mice, 0/1 L. crispatus-exposed mice). ‘E’ denotes areas of exfoliation. (TIF) [file ppat.1006238.s005.tif]

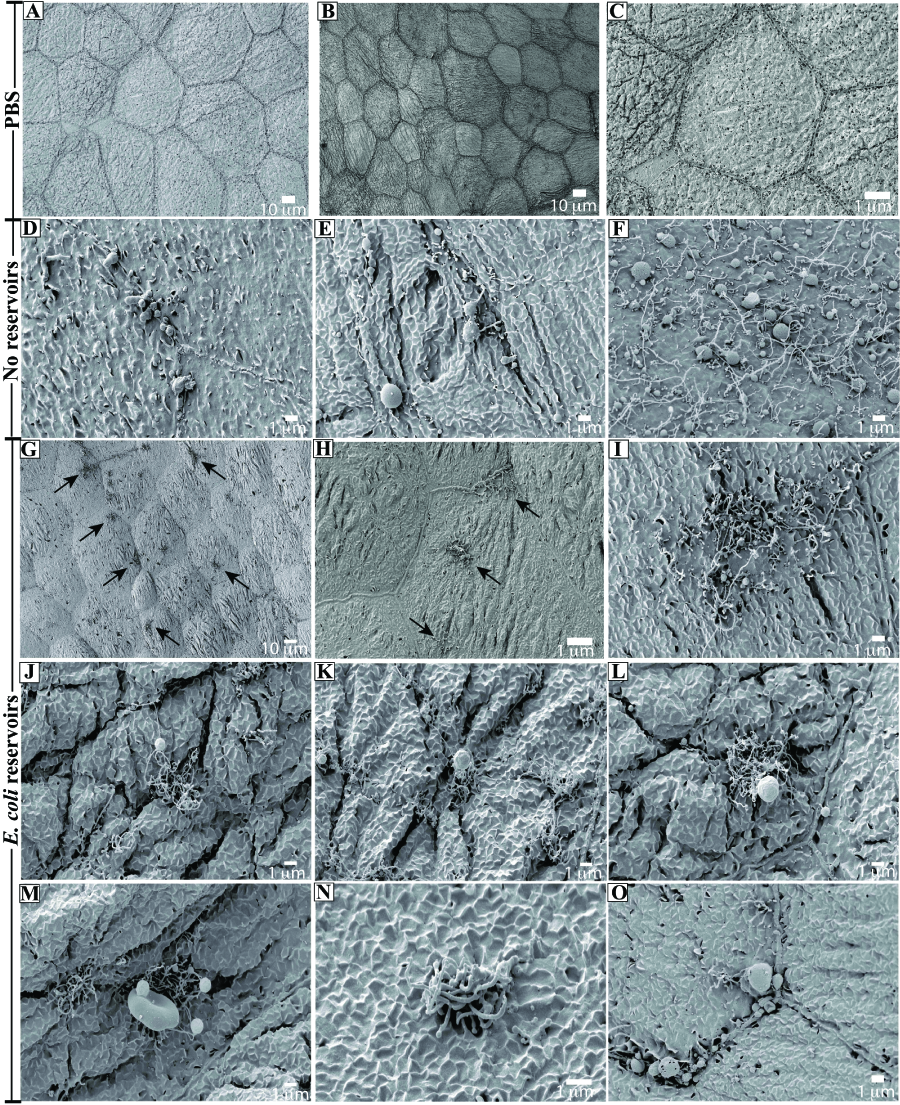

Supplement: S6 Fig — Scanning electron microscopy (SEM) of splayed bladders from control mice exposed to PBS (A-C), or from naïve (no E. coli reservoirs) (D-F) and E.coli reservoir-containing (G-O) mice exposed twice to G. vaginalis (12 h apart) and harvested 3 hours after the second exposure. Black arrows denote areas of membrane protrusions (~100 nm diameter) and blebbing. Panel M shows a red blood cell, seen in one bladder exposed to G. vaginalis. Images are representative of two independent experiments with mice containing E. coli reservoirs (phenotype was observed in 7/7 G. vaginalis-exposed mice and 0/3 PBS-exposed mice) and three independent experiments with naive mice (phenotype was observed in 7/7 G. vaginalis-exposed mice and 0/3 PBS-exposed mice). (TIF) [file ppat.1006238.s006.tif]

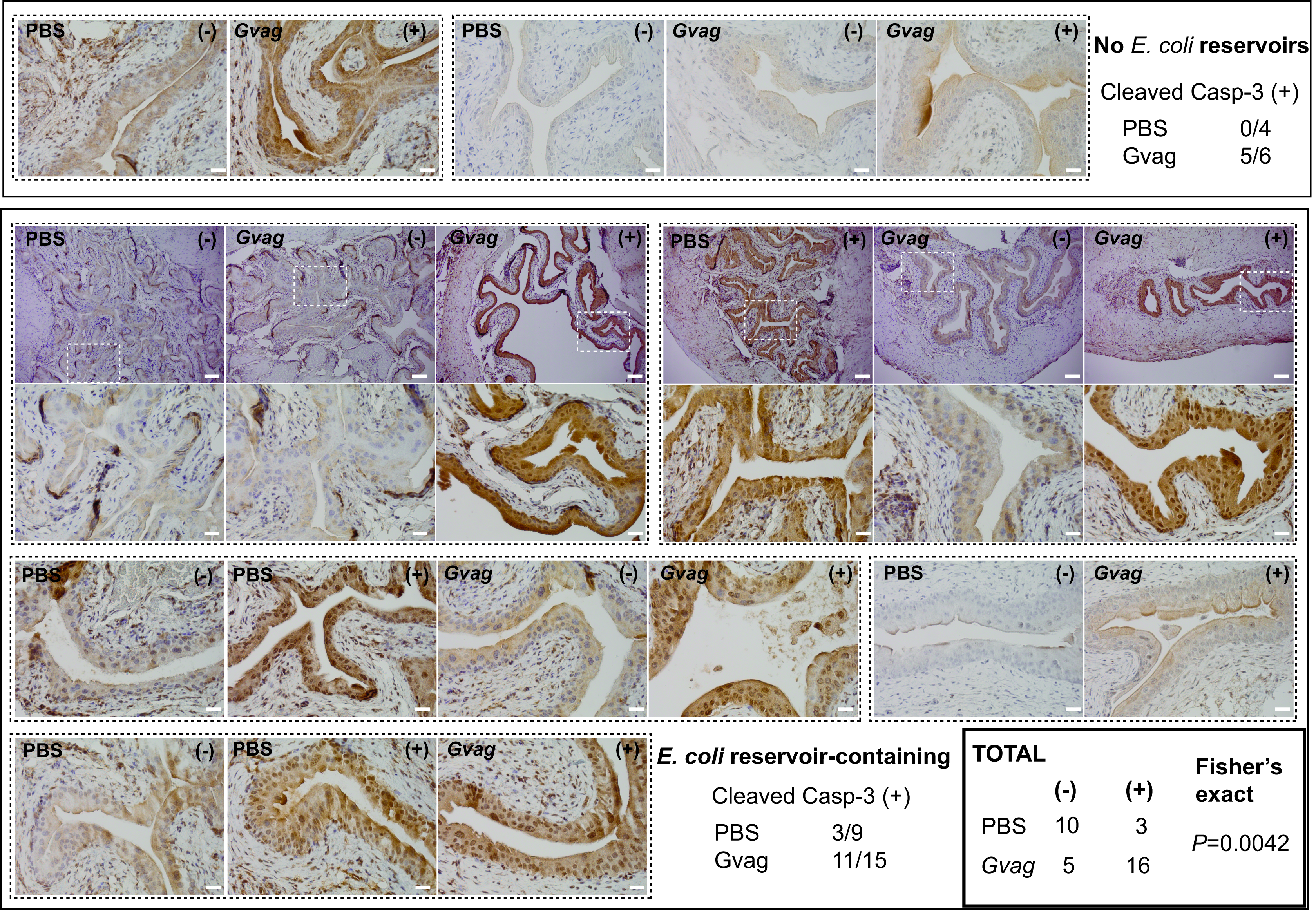

Supplement: S7 Fig — Additional images from experiments described in Fig 2C. Top box: bladders from naïve mice (no E. coli reservoirs). Bottom box: bladders from mice containing E. coli reservoirs. Images within each dotted line box are from bladder sections that were on the same slide. Scale bars are 20 μm, with the exception of the top row of images from E. coli reservoir-containing bladder are low magnification (scale bar 100 μm), with white dotted boxes denoting the area at higher magnification in the image directly below. Box in the bottom right corner shows the number of mice in each group that were either (-) or (+) for cleaved caspase-3 staining based on blinded scoring, as described in Materials and Methods. These values were used to generate the graph shown in Fig 2D. (TIF) [file ppat.1006238.s007.tif]

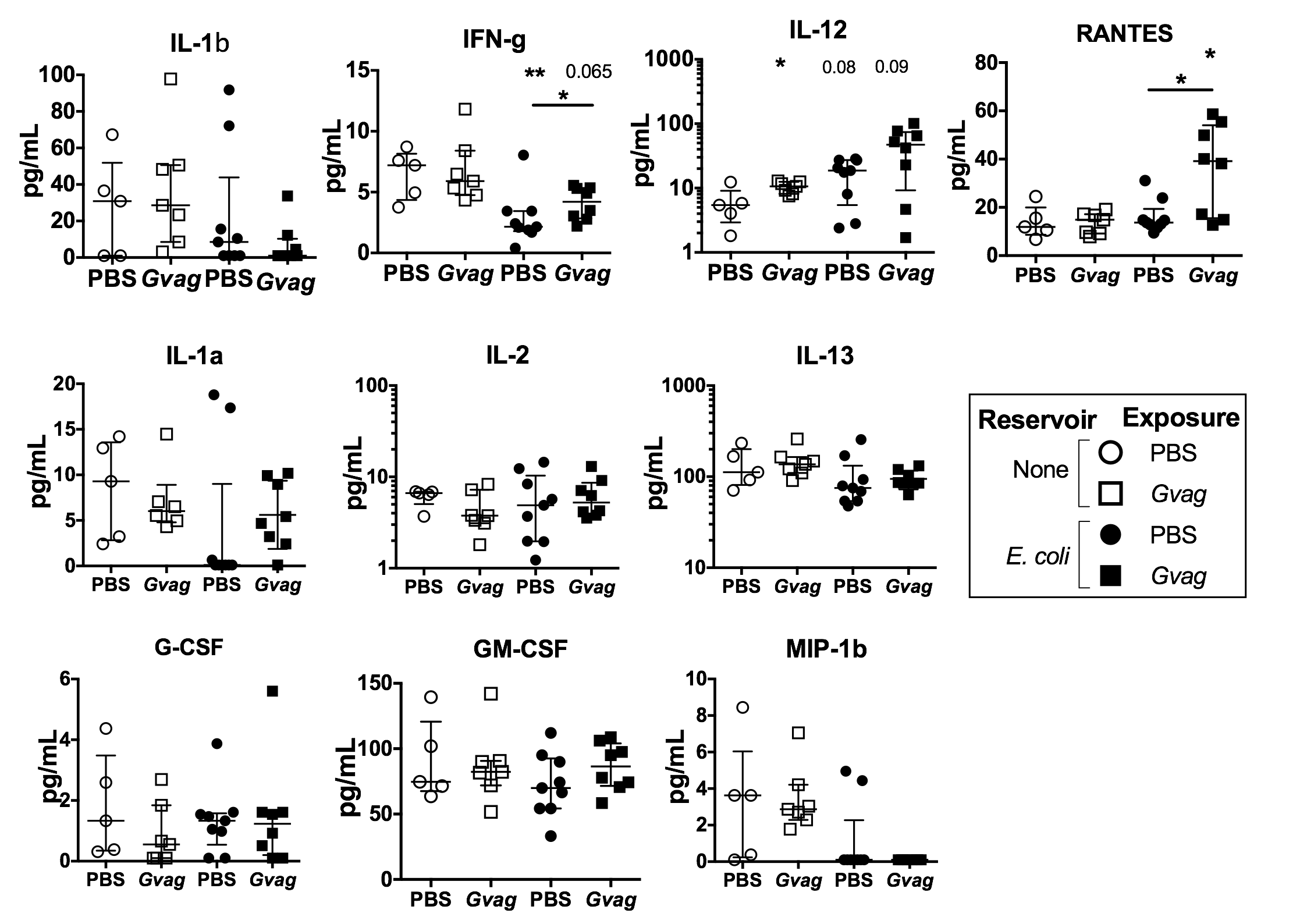

Supplement: S8 Fig — Naive mice (open symbols) or E. coli reservoir-containing mice (closed symbols) exposed to either PBS (circles) or G. vaginalis (squares). A Kruskal-Wallis test detected significant difference between the groups for RANTES, IL-12 and IFN-γ. A D’Agostino-Pearson omnibus normality test was performed, followed by appropriate post-hoc pairwise analysis (either unpaired t-test or Mann-Whitney U test). ** P = 0.01; * P < 0.05. Unless otherwise depicted with a line, P values represent statistically significant differences from naive mice exposed to PBS (open circles). (TIFF) [file ppat.1006238.s008.tiff]
